# A prediction model identifying glycolysis signature as therapeutic target for psoriasis

**DOI:** 10.3389/fimmu.2023.1188745

**Published:** 2023-05-02

**Authors:** Yanhong Shou, Ronghui Zhu, Zhenwei Tang, Xiao-Yong Man

**Affiliations:** ^1^ Department of Dermatology, Second Affiliated Hospital, Zhejiang University School of Medicine, Hangzhou, China; ^2^ Department of Dermatology, Huashan Hospital, Fudan University, Shanghai, China

**Keywords:** single-cell RNA seq, psoriasis, glycolysis, molecular signature, therapeutic effects

## Abstract

**Background:**

The hyperproliferation featured with upregulated glycolysis is a hallmark of psoriasis. However, molecular difference of keratinocyte glycolysis amongst varied pathologic states in psoriasis remain elusive.

**Objectives:**

To characterize glycolysis status of psoriatic skin and assess the potential of glycolysis score for therapeutic decision.

**Methods:**

We analyzed 345414 cells collected from different cohorts of single-cell RNA seq database. A new method, *Scissor*, was used to integrate the phenotypes in GSE11903 to guide single-cell data analysis, allowing identification of responder subpopulations. *AUCell* algorithm was performed to evaluate the glycolysis status of single cell. Glycolysis signature was used for further ordering in trajectory analysis. The signature model was built with logistic regression analysis and validated using external datasets.

**Results:**

Keratinocytes (KCs) expressing *SLC2A1* and *LDH1* were identified as a novel glycolysis-related subpopulation. Scissor^+^ cells and Scissor^−^ cells were defined as response and non-response phenotypes. In Scissor^+^ *SLC2A1^+^ LDH1^+^* KCs, ATP synthesis pathway was activated, especially, the glycolysis pathway being intriguing. Based on the glycolysis signature, keratinocyte differentiation was decomposed into a three-phase trajectory of normal, non-lesional, and lesional psoriatic cells. The area under the curve (AUC) and Brier score (BS) were used to estimate the performance of the glycolysis signature in distinguishing response and non-response samples in GSE69967 (AUC =0.786, BS =17.7) and GSE85034 (AUC=0.849, BS=11.1). Furthermore, Decision Curve Analysis suggested that the glycolysis score was clinically practicable.

**Conclusion:**

We demonstrated a novel glycolysis-related subpopulation of KCs, identified 12-glycolysis signature, and validated its promising predictive efficacy of treatment effectiveness.

## Introduction

Psoriasis, a chronic immune-mediated skin disorder occuring in approximately 2%–3% of the population worldwide, is characterized by raised scales and inflammatory eruptions on skin (1). Histologically, lesional skin is featured with abnormal proliferation of keratinocytes and is believed to be the main cause of clinical manifestations. Understanding why psoriatic keratinocytes being hyperproliferative remains critical for its treatment.

According to previous studies (2, 3), hyperproliferative keratinocytes in psoriatic lesions are incompletely differentiated and metabolically activated. In general, hyperproliferative cells require energic support to meet their self-accelerating cellular processes (2). A phenomenon termed “the Warburg effect” is well studied that cancer cells are dependent on glycolysis for energy production (4, 5). Glycolysis is a major source of energy generation, supporting rapid proliferation in many cells (6, 7). In psoriasis, skin cells undergo complete turnover within three or four days, whereas in healthy skin, this process takes one month (8). The pathologic proliferation of keratinocytes is one of the pathophysiological hallmarks of psoriasis (2). Previous research has indicated that enhanced glucose metabolism is essential for proliferating keratinocytes (9). Upregulated glucose transporter 1 (*GLUT1*) expression is correlated with increased Psoriasis Area and Severity Index (PASI) score, implying that it could be a target for abnormal hyperproliferation (10). Further research into glycolytic molecules linked to psoriasis pathogenesis is required.

Although metabolites can be directly determined by liquid chromatography-mass spectrometry technology, higher requirements of sample storage and easier degradation of targets are common. As a result, methods for determining the metabolic status via gene expression have been developed. Bulk RNA sequencing (bulk RNA-seq) represents the average of gene expression patterns at the whole population level, and the development of single-cell RNA sequencing (scRNA-seq) technologies allows the transcriptome profiling to be investigated at a single-cell resolution. By applying up-to-date single-cell sequencing, the metabolic reprogramming of single cell can even be identified via bioinformatic methods.

Here we collected scRNA-seq data from several recent studies (11–13) and analyzed the glycolysis level in diverse cell types, especially epidermal keratinocytes. Moreover, the phenotypes in GSE11903 were applied to recognize the specific subpopulations responsible for treatment. We sought to investigate the molecular signature and calculated the glycolysis score. According to the gene expression data and clinical information, an individual therapeutic effect assessment was performed. Before a long-term treatment regimen, gene expression studies could identify a potential therapeutic response at the molecular level.

## Methods

### Data acquisition

Single-cell transcriptome profiling from E-MTAB-8142 (11), which using skin biopsies from 24 patients with psoriasis (12 lesional samples, 12 non-lesional samples), and 40 healthy control subjects, was obtained via the European Bioinformatics Institute (https://www.ebi.ac.uk/). We also collected the scRNA-seq data including 5 patients with psoriasis and 3 healthy control subjects from GSE150672 (12) via the Gene Expression Omnibus (GEO) database (https://www.ncbi.nlm.nih.gov/geo/) and collected single-cell transcriptome profiling of normal and inflamed human epidermis from EGAS00001002927 (13) via the Genome-phenome Archive (EGA) database (https://ega-archive.org). RNA-seq datasets can be accessed on GEO: GSE30999 (14), GSE41664 (15), GSE78097 (16), GSE13355 (17), GSE14905 (18), GSE69967 (19), GSE11903 (20), GSE85034 (21).

### Data filtering and preprocessing

The following criteria were used to filter cells: (1) the total number of unique molecular identifiers (UMIs) per cell; (2) the number of detected genes per cell; and (3) the ratio of mitochondrial genes. The UMI count ranged from 200 to 50000, and the number of genes detected per cell ranged from 10% to 90% of total detected levels. High-quality cells were reserved if the proportion of mitochondrial genes was <10%. Doublets were detected by the package *DoubletFinder (* 22). Cells identified as doublets were excluded.

### Cell type recognition

Based on the top 15 principal components and the top 2000 variable genes, batch effects among the datasets were eliminated using the *RunHarmony* function (23). Uniform Manifold Approximation and Projection (UMAP) (24) with a resolution of 0.5 coordinate *FindAllMarkers* function in *Seurat* was then used for cluster-specific genes.

### Scissor selected cells

In the GSE11903 dataset of psoriasis treated with etanercept, we observed responders and non-responders. Combined with the phenotypes collected from GSE11903, the new approach *Scissor* (version 2.0.0) was executed to recognize the phenotype-related cells from single-cell data (25). Logistic regression with the parameter α 0.05 was set in the process. Scissor^+^ cells and Scissor^−^ cells will be linked to the responder and non-responder phenotypes, respectively.

### Pathway activity calculation

Pathway of glycolysis/gluconeogenesis was obtained from Kyoto Encyclopedia of Genes and Genomes (KEGG) database (http://www.genome.jp/kegg). According to the published articles, the major genes in the Th17/Th22 pathway were then defined and summarized. The *AUCell* package uses the “Area Under the Curve” (AUC) to calculate the activity level of gene sets using a rank-based scoring method and computes a gene set activation score for each cell. Following that, the *AUCell* package (version 1.8.0) (26) was used to calculate the activity of glycolysis/gluconeogenesis or the Th17/Th22 pathway for individual cells.

### Differential gene expression analysis

We performed a differential gene expression analysis on a per cluster of keratinocytes for lesional vs non-lesional psoriasis samples, then retained differentially expressed genes (DEGs) with adjusted P value < 0.05 & abs avg_log2FC >0.25. DEGs were extracted, and further functional enrichment analysis was carried using the *clusterProfile* package (version 3.18.1) (27). Based on the metabolic pathways from KEGG database, package *fgsea* (version 1.16.0) was performed to calculate the normalized enrichment score (28).

### Single-cell trajectory analysis

We were interested in the role of glycolysis/gluconeogenesis in keratinocytes, thus focusing on downstream extraction and visualization of keratinocytes. Package *monocle* (version 2.6.4) (29) was utilized to construct pseudotime trajectories. Overlapped genes between DEGs and glycolysis/gluconeogenesis gene set were determined as gene signature and then were used for ordering in a semi-supervised manner.

### Calculation of gene signature score

Overlapped genes were identified between DEGs and the glycolysis/gluconeogenesis gene set, and logistic regression was performed using the *glm* function. To ensure the algorithm was robust, well-correlated genes were selected in the model (P<0.05). Furthermore, we used the following method to calculate each patient’s signature score: score=k∑gene. In this formula, “gene” refers to the gene expression level of each gene signature, and “k” refers to the coefficient for each gene signature.

### Scoring classifier

To estimate and visualize the performance of the gene signature score, the Receiver Operating Characteristic Curve (ROC) was depicted and the Area Under the Curve (AUC) was calculated. Calibration curves were depicted for calibration visualization. Decision curve analysis (DCA) was conducted to assess the clinical benefits. *pROC* (version 1.18.0), *rms* (version 6.2-0), and *rmda* (version 1.6) were used in this part.

### Statistical analysis

Significance between non-responders versus responder phenotype was compared through the t-test. The statistical difference between normal, non-lesional, and lesional groups was analyzed using *ANOVA* approaches. Spearman’s correlation coefficients were calculated using the *corrplot* package (version 0.84). Our analyses were performed with R software, R version 4.0.5.

## Results

### Single-cell RNA-seq profiling and screening of marker genes

**Figure 1:**
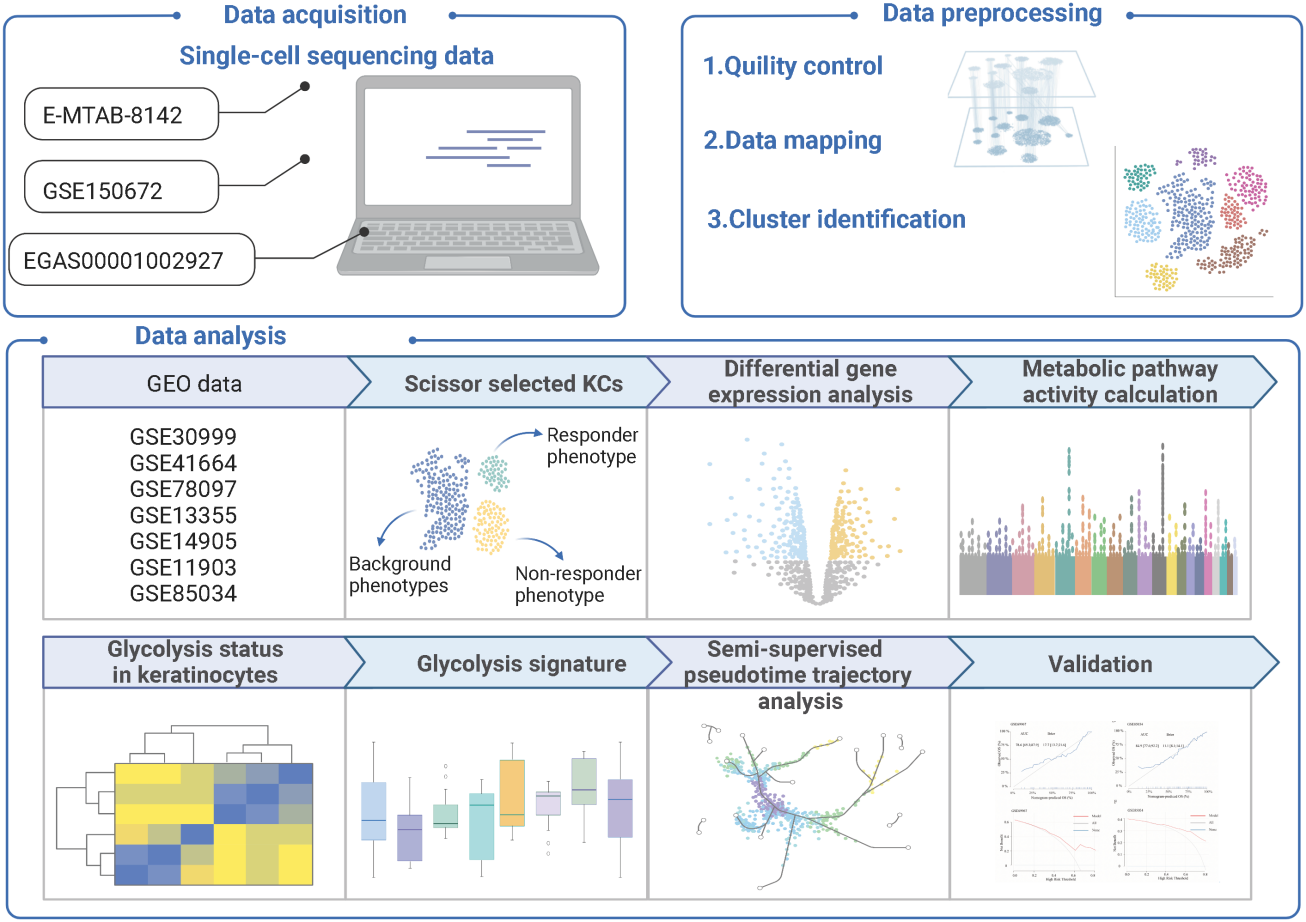
The flowchart of the research.

**Figure 2:**
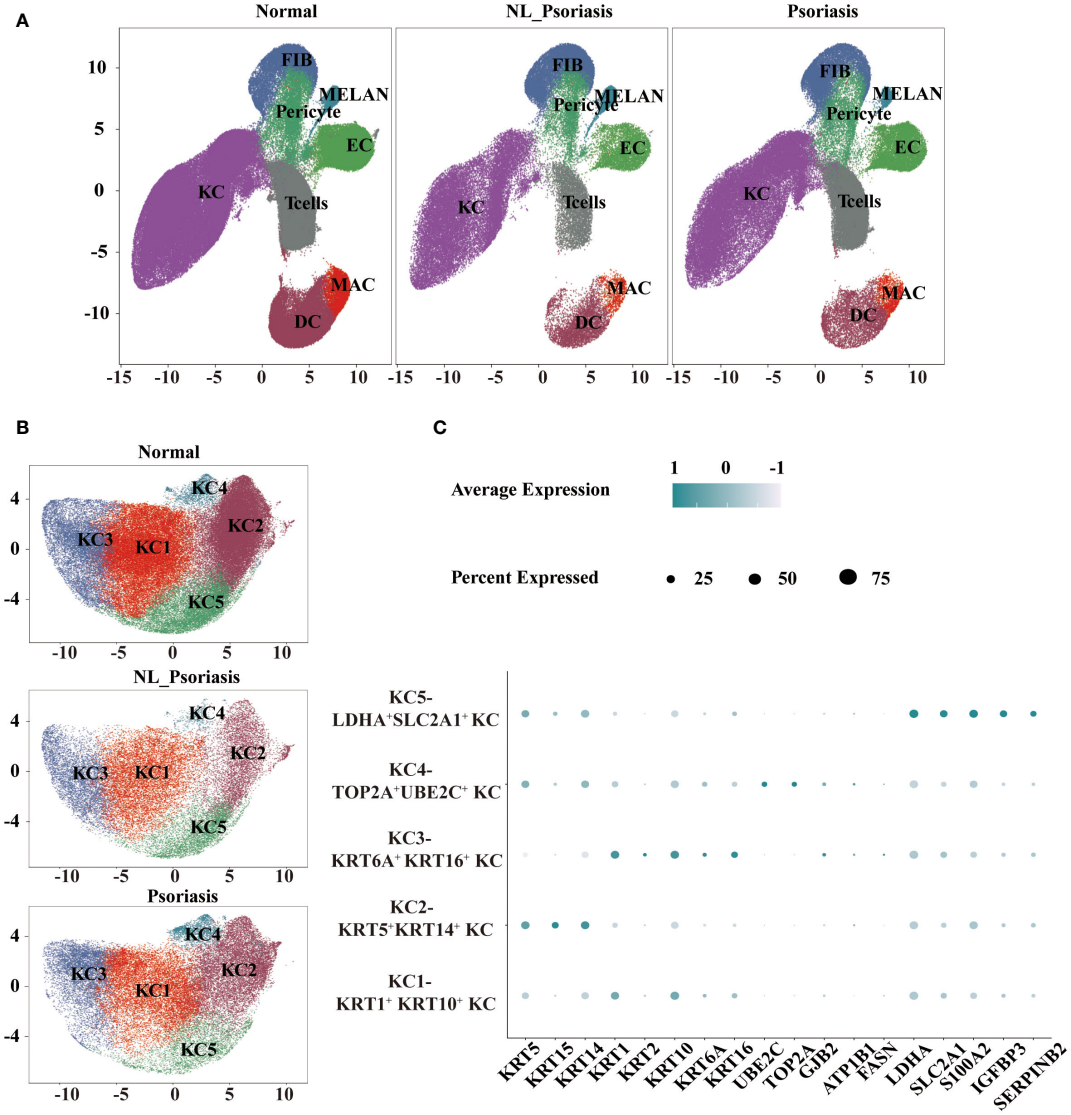
Single-cell landscape of normal and psoriasis human skin. **(A)** UMAP visualization of eight cell types. **(B)** UMAP representation of five clusters of keratinocytes. **(C)** A dot plot displaying critical marker transcripts used to distinguish keratinocytes. UMAP, Uniform manifold approximation and project.

The study workflow was shown in **Figure 1**. We integrated the selected datasets using *Seurat*’s standard workflow and identified 201915, 59700, and 83799 cells from normal, non-lesional psoriasis, and lesional psoriasis samples, respectively. Using graphical unsupervised clustering, we recognized seven clusters of cells (**Figure 2A**) and defined them according to the signature expression of marker genes (**Supplementary Figure 1A**).

First-level analysis showed that keratinocytes (KCs) were recognized by *KRT* genes. T cells were distinguished by coordinate upregulation of *CD3D*, *CD3G*, and *CD3E*, and macrophages/dendritic cells (MAC/DC) showed high *HLA* levels. Fibroblasts (FIB) highly showed expressions of *COL1A1*, *DCN*, and *LUM*. Finally, endothelial cells (EC) were recognized by the *VWF* and *PECAM1* levels. Melanocytes (MELAN) indicated elevated for transcripts known to be expressed in melanocyte pigment synthesis pathway. A cluster of cells expressed *ACTA2* and *TAGLN* were recognized as pericytes. We determined the relative proportion of subpopulations in all samples, suggesting increased T cells in lesional psoriasis versus in normal skin (P<0.05, **Supplemental Figure 1B**).

To further characterize KC, we performed the second-level clustering analysis of them. Based on UMAP (**Figure 2B**), five subpopulations were identified: ‘KC1’, ‘KC2’, ‘KC3’, ‘KC4’, and ‘KC5’. As shown in **Figure 2C**, ‘KC1’ expressed high levels of *KRT1* and *KRT10* and corresponded to suprabasal keratinocytes (*KRT1^+^KRT10^+^* KC). ‘KC2’ were defined as basal keratinocytes for their high expression levels of *KRT5* and *KRT14* (*KRT5^+^ KRT14^+^* KC). Due to the significant expression of *KRT1*, *KRT10*, *KRT6A*, and *KRT16*, a cluster of KC3 were recognized as inner root sheath (IRS)-sebaceous cells (*KRT6A^+^KRT16^+^* KC). And the UBE2C - and TOP2A -expressing KC4 were annotated as proliferating keratinocytes (*TOP2A^+^UBE2C^+^* KC). Genes involved in glycolysis and inflammation (*LDH1*, *SCL2A1*, *S100A2*, *IGFBP3*, and *SERPINB2*) were highly expressed in the ‘KC5’, leading us to define them as a glycolysis-related subpopulation (*LDHA^+^SLC2A1^+^* KC).

### Identifying the responder and non-responder keratinocyte subpopulations

**Figure 3:**
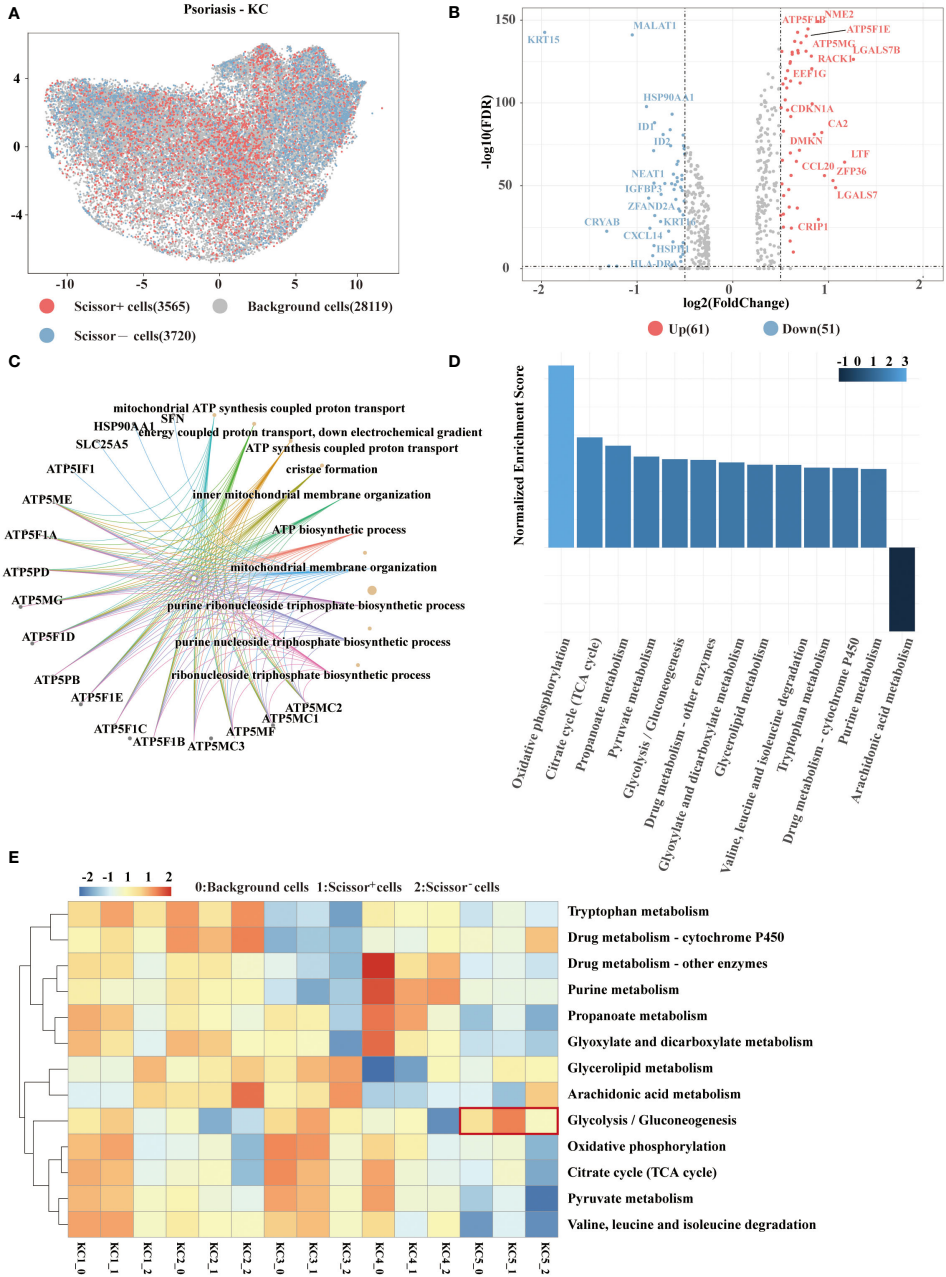
Scissor identification results on psoriasis keratinocytes. **(A)** UMAP visualization of the Scissor-selected cells. The red and blue dots show Scissor^+^ and Scissor^−^ cells, representing responder and non-responder phenotypes, respectively. **(B)** Volcano plot of differential gene expressions in Scissor^+^ cells versus Scissor^−^ cells. **(C)** GO enrichment analysis of differential gene expressions between Scissor^+^ cells and Scissor^−^ cells. **(D)** GSEA plot of the up and down metabolic pathways. The adjusted P value <0.05. **(E)** Heat map of enriched metabolic pathways. The red and blue elements suggest the activated and repressed pathways in keratinocytes. GO, gene ontology; GSEA, Gene set enrichment analysis.

Application of biologics have exhibited favorable results in psoriasis therapy but also showed varied responses in some patients. We analyzed clinical phenotypical features provided by a psoriasis dataset to investigate the mechanism underlying different responses. *Scissor* analysis was performed on 35405 keratinocytes from psoriasis samples. A total of 3565 Scissor^+^ cells associated with the responder phenotype and 3720 Scissor^−^ cells related to the non-responder phenotype were recognized (**Figure 3A**). To characterize the transcriptional features, 61 upregulated genes and 51 downregulated genes were differentially expressed in Scissor^+^ cells versus Scissor^−^ cells (**Figure 3B**). Notably, we found that numerous ATP-related genes were among the overexpressed genes listed above. Consistently, several ATP synthesis pathways were enriched in Scissor^+^ cells through GO enrichment analysis (**Figure 3C**). ATP production includes three important cellular processes——glycolysis, oxidative phosphorylation, and beta-oxidation. By performing Gene Set Enrichment Analysis (GSEA), we confirmed the significant metabolic pathways based on the ranked matrix of putative differentially expressed genes (**Figure 3D**). In 85 metabolic pathways, the top five were oxidative phosphorylation, the TCA cycle, propanoate metabolism, pyruvate metabolism, and glycolysis.

*AUCell* was used to calculate metabolic pathway activity in Scissor^+^ and Scissor^−^ cells to evaluate their metabolic status. As shown in the heatmap (**Figure 3E**), the glycolysis pathway was especially intriguing in *LDHA^+^SLC2A1^+^* KC. In addition, the glycolysis score of Scissor^+^ cells were significantly higher than that of Scissor^−^ cells and other background cells, revealing an important role of glycolysis in response to biologic psoriasis treatment (P=0.017).

### Estimation of glycolysis in keratinocytes

**Figure 4:**
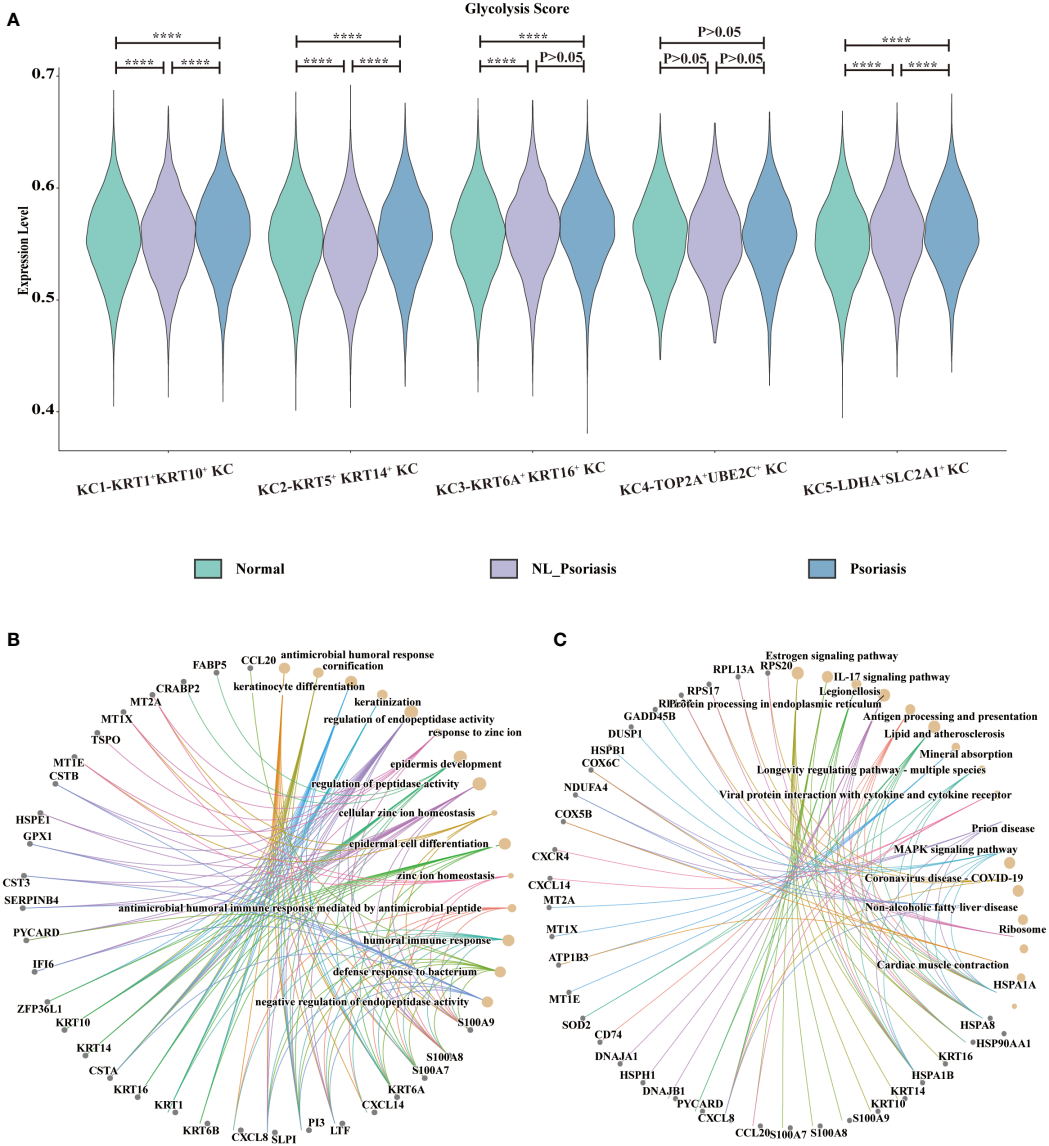
Glycolysis status in keratinocytes. **(A)** Display of glycolysis for subpopulations. Y-axis corresponds to glycolysis pathway activity. The X-axis represents KC population in normal skin, non-lesional and lesional psoriasis samples. **(B, C)** GO and KEGG enrichment analysis of top-100 differentially expressed genes between non-lesional phenotype and lesional phenotype. GO, gene ontology; KEGG, Kyoto Encyclopedia of Genes and Genomes ****P<0.0001.

The glycolysis status in keratinocytes was observed. In comparison with normal samples, the glycolysis score was significantly higher in psoriasis samples in *KRT5^+^ KRT14^+^* KC, *KRT1^+^KRT10^+^* KC, and *LDHA^+^SLC2A1^+^* KC (all P<0.001, **Figure 4A**). Next, combined with the above-observed specific makers of glycolysis in *LDHA^+^SLC2A1^+^* KC, we suspected that glycolysis stands out.

To investigate the gene signature of lesional phenotype, differential gene expression analysis was performed between non-lesional and lesional samples. The top 100 upregulating DEGs in lesional *LDHA^+^SLC2A1^+^* KC were enriched in biological processes related to keratinocyte differentiation, cornification, keratinization, and epidermis development (**Figure 4B**), as well as the KEGG pathway of IL-17 signaling (**Figure 4C**).

### Pseudo−time trajectory reconstruction

**Figure 5:**
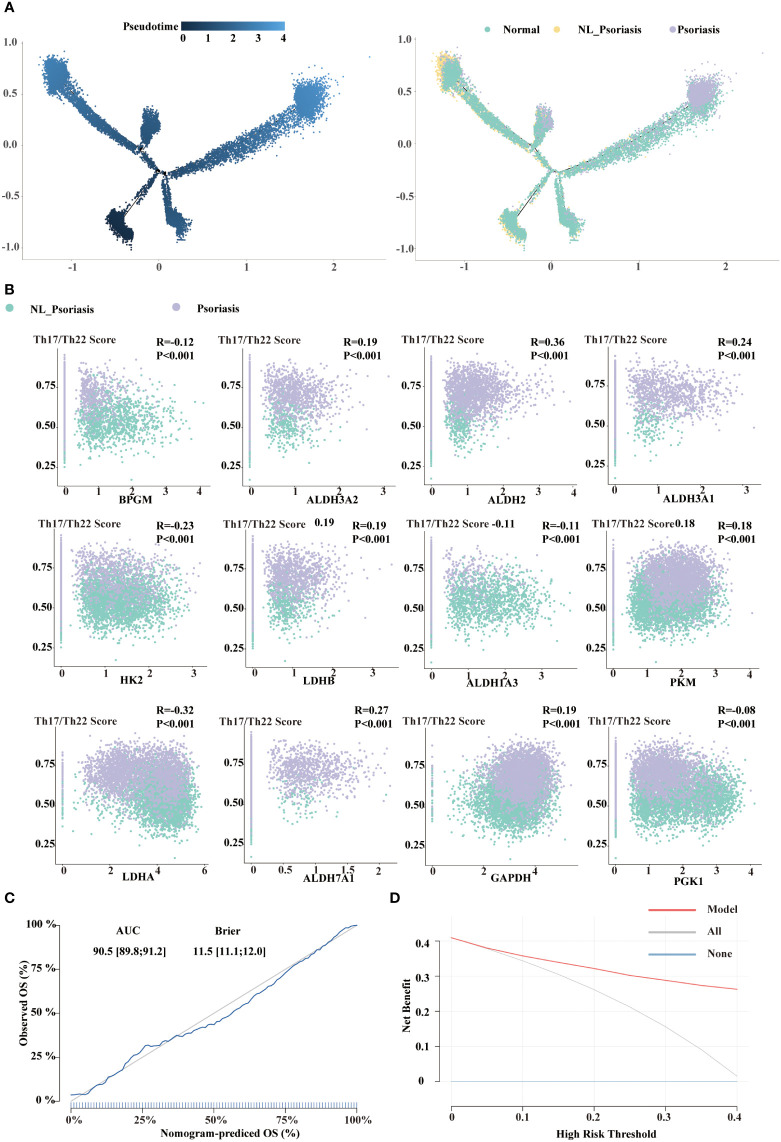
Construction of 12-gene signature. **(A)** Distribution of KC5 on the pseudo-time trajectory in a semi-supervised manner. Cells are colored based on pseudotime and tissue types. **(B)** The correlation between Th17/Th22 pathway activity and the selected signatures. **(C)** Calibration curve of the 12-gene signature model. **(D)** Decision Curve Analysis of the 12-gene signature model.

To focus on glycolysis in developmental decision-making within individual cells in disease progression, we identified 12 overlapped genes (*BPGM*, *ALDH3A2*, *ALDH2*, *ALDH3A1*, *HK2*, *LDHB*, *ALDH1A3*, *PKM*, *LDHA*, *ALDH7A1*, *GAPDH*, and *PGK1*) between the DEGs and glycolysis gene set. Pseudotime trajectory analysis in a semi-supervised manner was executed. Cells from normal samples were mainly at the start of the projected timeline trajectory, while cells from non-lesional and lesional psoriasis samples were positioned in the middle and the end, respectively (**Figure 5A**). These results highlighted that glycolysis gene regulation coordinates trajectory of disease progression in keratinocytes.

Correlation analysis suggested that mRNA expression of overlapped genes and Th17/Th22 score were correlated across a variety of ranges from R= -0.29 to 0.37 (all P<0.001, **Figure 5B**). Eight were positively associated with the lesional phenotype, whereas eight were negative with the non-lesional phenotype. This minimum set of genes indicated candidate targets for further investigation and therapy for psoriasis.

Multivariate logistic regression analysis was performed on these 12-glycolysis gene signature, and a glycolysis-related model was established. The model performed well in distinguishing between non-lesional and lesional psoriasis samples. The model had a good discrimination for distinguishing non-lesional and lesional psoriasis samples (AUC=0.905, 95%CI 0.898-0.912; BS=11.5, 95%CI 11.1-12.0). The calibration curve showed the agreement between the observed and expected numbers predicted by the model (**Figure 5C**). Finally, we performed decision curve analysis to assess the clinical impact of the model. As shown in **Figure 5D**, the decision curve suggested the clinical net benefit of the use of our glycolysis-related model if the threshold probability is over 0.05.

### Glycolysis gene signature score to evaluate the clinical changes

**Figure 6:**
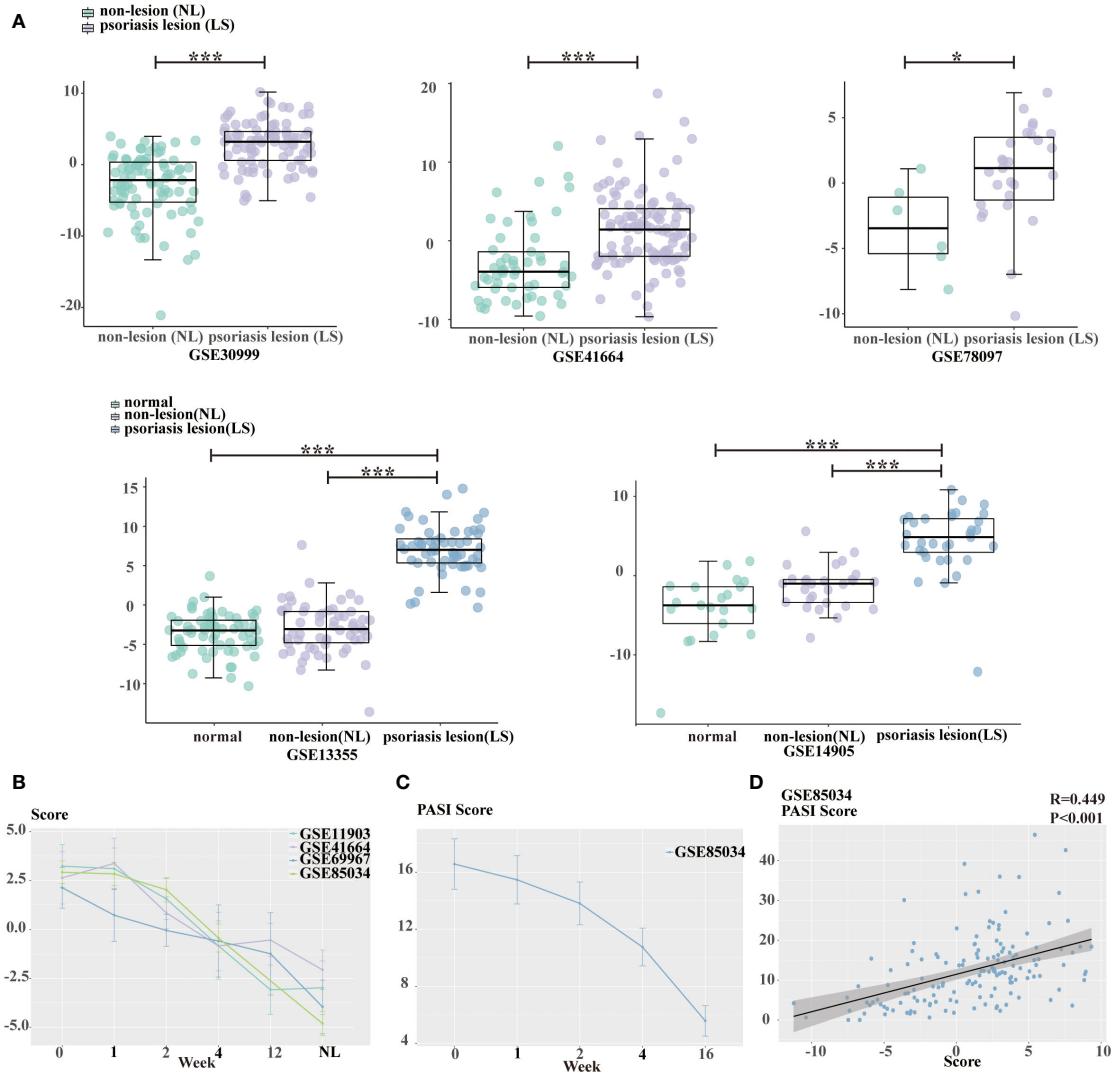
Estimation of glycolysis score. **(A)** Boxplot presenting the difference of glycolysis score among healthy skin, non-lesional psoriasis samples, and lesional psoriasis samples in GSE30999, GSE41664, GSE78097, GSE13355, and GSE14905. **(B)** The declining tendency of glycolysis score with treatment time in GSE11903, GSE41664, GSE69967, and GSE85034 cohorts. **(C)** The decreasing tendency of PASI scores with treatment time in GSE85034 cohort. **(D)** The correlation between glycolysis score and PASI score in GSE85034 cohort. *P<0.05, ***P<0.001.

To further examine the clinical relevance of the above signatures, we chose six independent psoriasis datasets obtained from GEO and performed score calculation using the 12 genes. In comparison to healthy and non-lesional psoriasis samples, lesional psoriasis samples has highest scores (all P<0.05, **Figure 6A**). Moreover, there was a progressive decline of signature scores over time during treatment, including etanercept, tofacitinib, and methotrexate (MTX) (**Figure 6B**).

In the dataset GSE85034, we observed a time-dependent and concomitant decrease in both signature score and PASI score (**Figure 6C**). Furthermore, spearman’s correlation analysis showed that PASI clinical score was positively associated with the glycolysis gene signature score (R=0.449, **Figure 6D**).

### Glycolysis gene signature score to assess the therapeutic efficacy

**Figure 7:**
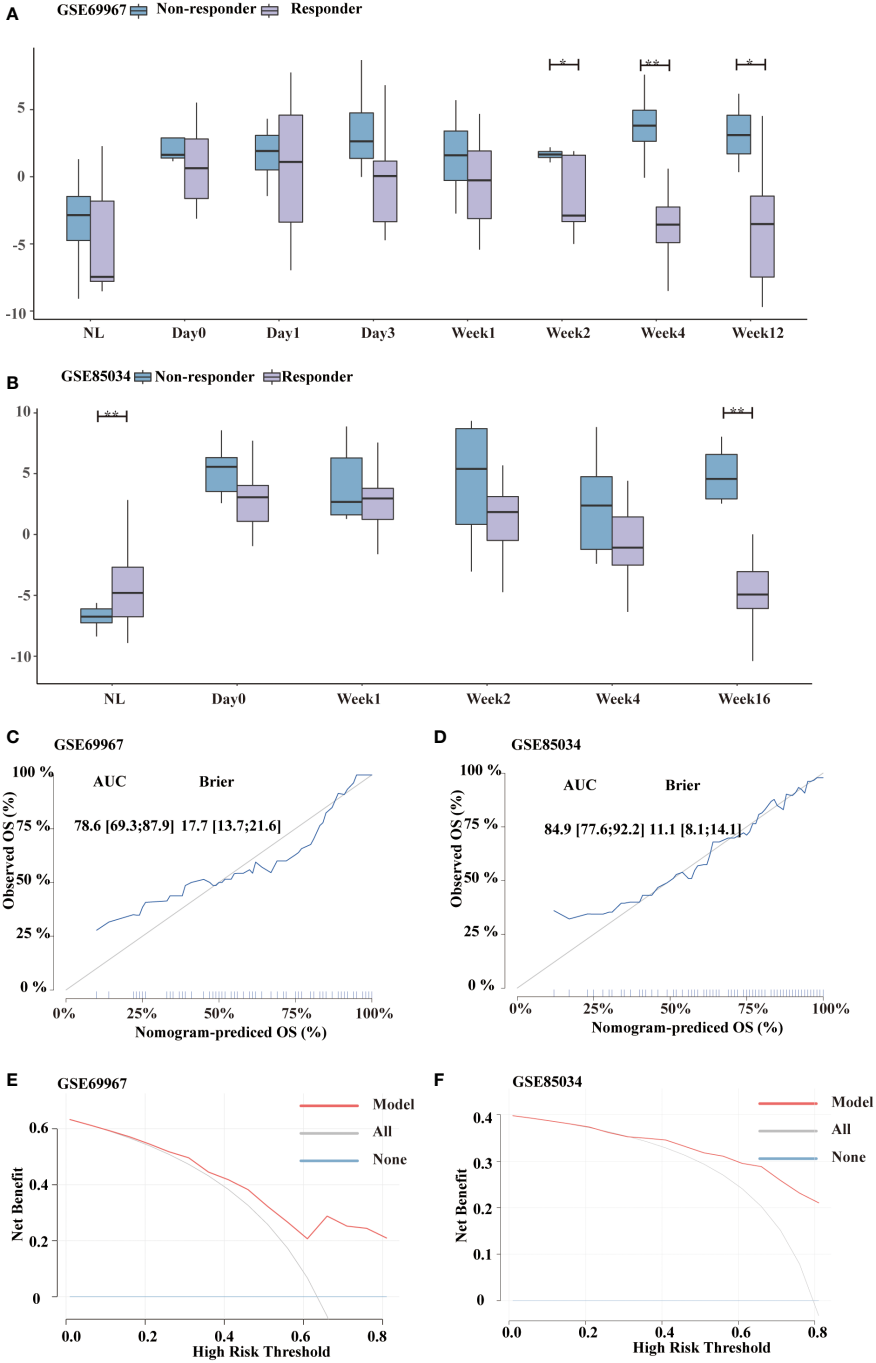
Distinguishment of responders and non-responders using glycolysis score. **(A, B)** Boxplot showing the distribution of glycolysis score between responders and non-responders in GSE69967 (patients treated with tofacitinib) and GSE85034 (patients treated with methotrexate or adalimumab) cohort, respectively. **(C, D)** Calibration curve of the glycolysis signature in GSE69967 and GSE85034 cohort. **(E, F)** Decision Curve Analysis of the glycolysis signature in GSE69967 and GSE85034 cohort. *P<0.05, **P<0.01.

Non-responders and responders differed significantly in glycolysis score from week 2 forward for patients treated with tofacitinib in the GSE69967 dataset (**Figure 7A**). Furthermore, the decline in glycolysis score was more pronounced in responders than in non-responders. Similar results were observed in GSE85034 dataset, which included patients receiving methotrexate or adalimumab (**Figure 7B**). At week16, the difference between non-responders and responders was statistically significant. Next, we sought to investigate whether glycolysis score could predict future treatment response.

To differentiate the non-responders and responders, calibration curve analyses showed the diagnostic accuracy as follows: GSE69967 (AUC=0.786, 95%CI 0.693-0.879; BS=17.7, 95%CI 13.7-21.6, **Figure 7C**) and GSE85034 (AUC=0.849, 95%CI 0.776-0.922; BS=11.1, 95%CI 8.1-14.1, **Figure 7D**), showing that this model is still valid. In addition, we validated the clinical practicability using DCA (**Figures 7E, F**). These findings demonstrated the net benefit of the glycolysis model.

## Discussion

As glucose is a preferred bioenergetic substrate for proliferating cells, glucose uptake and utilization are essential in the pathogenesis of psoriasis. Based on the single-cell profiles of psoriasis, we report for the first-time status of intracellular glycolysis in a specific population of keratinocytes and identified a 12-gene prediction model. The validation of the model paved the way for distinguishing different tissues, classifying responders and non-responders, and predicting the effectiveness of therapy.

In our study, the expression of *SCL2A1* and *LDH1* was used to define a population of glycolysis-related keratinocytes. SLC2A1, one of the glucose transporters, is overexpressed in proliferating inflammatory cells and keratinocytes. In several models, SLC2A1 deletion attenuates inflammatory infiltration (30, 31). According to recent reports, SLC2A1 plays role in UV-irradiated mouse skin (32), during wound healing responses (33), and in psoriasis (10). LDH1 inhibition has been shown to reduce the damaging inflammatory contributions in rheumatoid arthritis and osteoarthritis (34, 35). In addition to expressing *SCL2A1* and *LDH1*, *LDHA^+^SLC2A1^+^* KC exhibited high levels of *S100A2*, *IGFBP3*, and *SERPINB2*, denoting inflammatory conditions in these keratinocytes.

Several methods have been proposed to identify disease-relevant cells from single-cell data, which helps to understand the pathogenic mechanisms. HoneyBadger (36) was carried out to recognize cancerous cells. *Scissor* was developed for discerning phenotype-specific cell subpopulations using phenotype information from bulk data (37). In our research, we introduce treatment response as a phenotype to infer phenotype-relevant cells from single-cell data. ATP production performed particularly specific in Scissor^+^ cells, and oxidative phosphorylation, TCA cycle, propanoate metabolism, pyruvate metabolism, and glycolysis were also enriched. In psoriasis, ATP synthesis demand may be a hallmark of therapy-responding keratinocytes.

Most of these enrichment patterns of DEGs were consistent with previous studies, including epidermal cell differentiation and the IL-17 signaling pathway. The results outlined here demonstrated changes in mRNA gene expression pointing to the progression of psoriasis. Furthermore, an upward trend of glycolysis was observed from healthy samples to lesional psoriasis samples in keratinocytes. These results indicated a significant global shift in glycolysis score from normal to psoriasis and the altered glycolysis level might play an essential role during psoriasis initiation and progression.

A total of 12 common transcriptome signature were defined. Note that not only the current signature is relevant for Th17/Th22 pathway activity, but also involved in the classification in the multivariable model. Furthermore, *monocle* can be performed to define and recover biological progression between cellular states, including differentiation, proliferation, and reprogramming (29). Using a semi-supervised algorithm based on these 12 genes, a branch of healthy cells was separated from a two-phase keratinocyte differentiation trajectory. Taken together, pseudotime trajectory analysis reveals the significance of glycolysis in disease progression.

Skin samples from psoriatic patients witness significant metabolic reprogramming, which is closely linked to phenotypic variation and progression. According to studies based on metabolomics, metabolites such as choline, glutamic acid, lactic acid, urocanic acid, and saturated fatty acids have been identified as psoriasis biomarkers (38, 39). The levels of amino acids were also associated with the severity of psoriasis and the effects of anti-TNFα treatment (40). Notedly, the glycolysis pathway in our research appeared to be more intrigued and performed especially higher level in Scissor^+^ cells than other cells. As a result, the metabolic shift reflects not only the pathologies of the disease but also the therapeutic response.

Here, different responses to diverse therapies were observed, including etanercept, tofacitinib, and MTX. We observed a decreasing glycolysis score over time, indicating that the glycolysis score can be viewed as an evaluation approach. Rather than being used as a predictive index, the glycolysis score can help discriminate between responders and non-responders. After receiving therapy with tofacitinib or MTX, responders had a greater decrease in glycolysis scores and a more favorable therapeutic profile. The glycolysis score assists in determining whether psoriasis patients are receptive to treatment, hence avoiding potential side effects and lowering expenditures. Our research sheds new insight on the utilization of an individual’s molecular data to create a customized treatment.

There are some limitations. Although we present a comprehensive transcriptome profile of glycolysis level analysis and identified significant genes, all our findings were based on public data sets and lacked some validation by experiments. Second, the biases from the retrospective studies might be inevitable, but we validated the results in several databases and demonstrated the reliability to a certain extent. Third, we have restricted our study to keratinocyte glycolysis. Hexokinase activity in dendritic cells has been linked to IL-23 and psoriasis-like inflammatory responses (41). It is imperative to conduct further research to investigate whether other cell types, particularly immune cells, exhibit altered glycolysis status.

We demonstrated characteristic changes in the glycolysis level in psoriasis across all available single-cell data, implying glycolysis status may be associated with disease severity and therapeutic response. Furthermore, we investigated specific glycolytic markers and validated their diagnostic and prognostic efficacy in the five cohorts. Glycolysis score used for prediction of treatment outcome may further benefit psoriasis populations.

## Data availability statement

The original contributions presented in the study are included in the article/**Supplementary Material**. Further inquiries can be directed to the corresponding author/s.

## Author contributions

Data curation and Formal Analysis, YS and RZ; Supervision, ZT; Writing – original draft, YS; Supervision and Writing – review & editing, X-YM. All authors contributed to the article and approved the submitted version.

## Supplementary material

Supplementary Figure 1
**(A)** Dot plot displaying the specific marker genes selected to classify subpopulations. **(B)** Plot of the proportion of cells in different subpopulations from normal, non-lesional psoriasis samples, and lesional psoriasis samples.
