# Supplementary figures and images for: A prediction model identifying glycolysis signature as therapeutic target for psoriasis
Source: Front Immunol. 2023 May 2;14:1188745. doi: 10.3389/fimmu.2023.1188745 (PMC10185821; doi:10.3389/fimmu.2023.1188745)

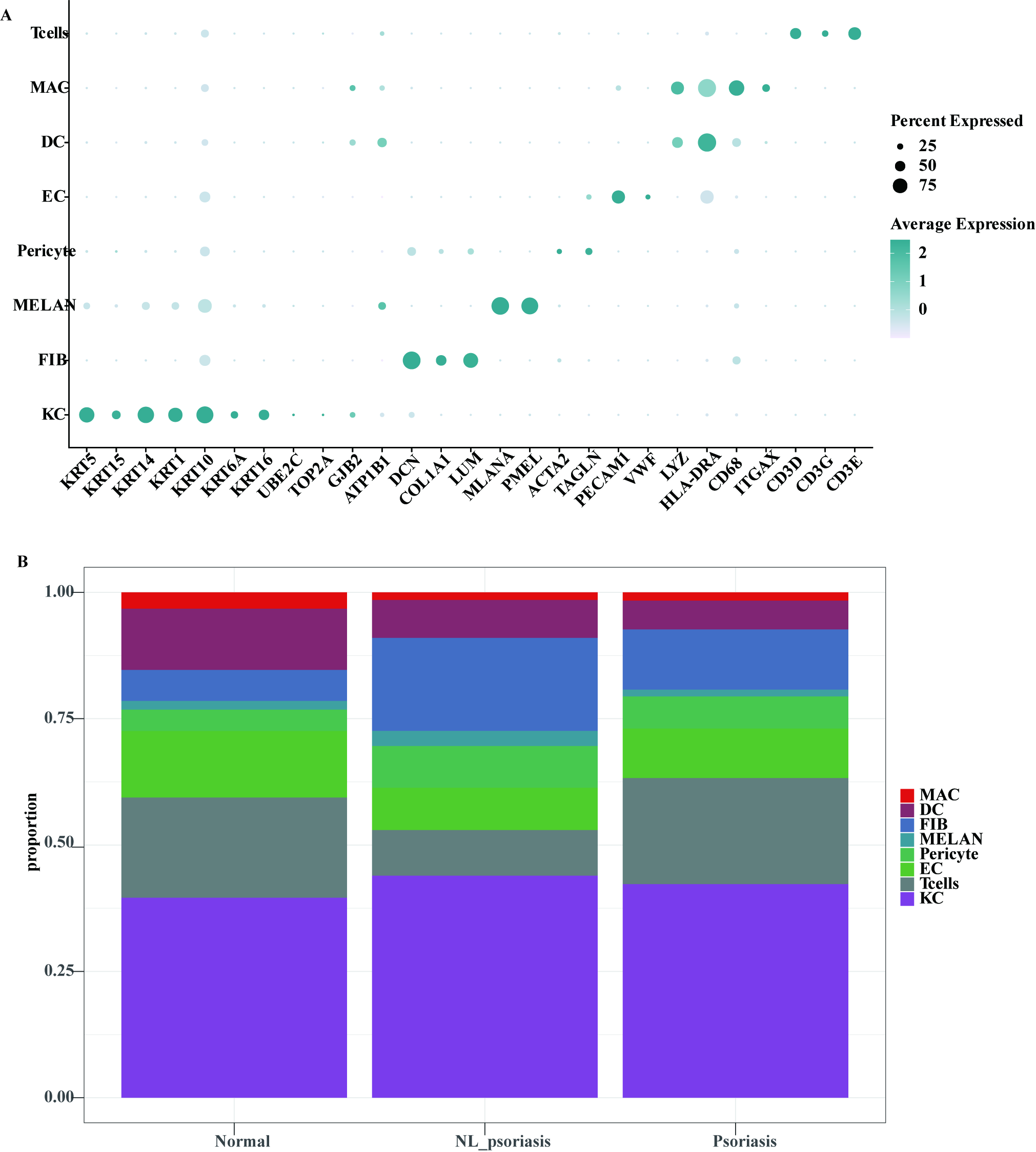

Supplement: Supplementary Figure 1 — (A) Dot plot displaying the specific marker genes selected to classify subpopulations. (B) Plot of the proportion of cells in different subpopulations from normal, non-lesional psoriasis samples, and lesional psoriasis samples. [file Image_1.tif]
